# Two Forkhead transcription factors regulate cardiac progenitor specification by controlling the expression of receptors of the fibroblast growth factor and Wnt signaling pathways

**DOI:** 10.1242/dev.122952

**Published:** 2016-01-15

**Authors:** Shaad M. Ahmad, Pritha Bhattacharyya, Neal Jeffries, Stephen S. Gisselbrecht, Alan M. Michelson

**Affiliations:** 1 Department of Biology, Indiana State University, Terre Haute, IN 47809, USA; 2 The Center for Genomic Advocacy, Indiana State University, Terre Haute, IN 47809, USA; 3 Laboratory of Developmental Systems Biology, National Heart Lung and Blood Institute, National Institutes of Health, Bethesda, MD 20892, USA; 4 Office of Biostatistics Research, National Heart Lung and Blood Institute, National Institutes of Health, Bethesda, MD 20892, USA; 5 Division of Genetics, Department of Medicine, Brigham and Women's Hospital and Harvard Medical School, Boston, MA 02115, USA

**Keywords:** Forkhead domain transcription factors, Signaling pathway receptors, Fibroblast growth factor receptor, Wnt signaling pathway receptors, Gene regulation, Cardiac progenitor specification, Cardiac mesoderm specification, Heart development, Cardiogenesis, Organogenesis

## Abstract

Cardiogenesis involves the coordinated regulation of multiple biological processes by a finite set of transcription factors (TFs). Here, we show that the Forkhead TFs Checkpoint suppressor homologue (CHES-1-like) and Jumeau (Jumu), which govern cardiac progenitor cell divisions by regulating Polo kinase activity, play an additional, mutually redundant role in specifying the cardiac mesoderm (CM) as eliminating the functions of both Forkhead genes in the same *Drosophila* embryo results in defective hearts with missing hemisegments. This process is mediated by the Forkhead TFs regulating the fibroblast growth factor receptor Heartless (Htl) and the Wnt receptor Frizzled (Fz): *CHES-1-like* and *jumu* exhibit synergistic genetic interactions with *htl* and *fz* in CM specification, thereby implying that they function through the same genetic pathways, and transcriptionally activate the expression of both receptor-encoding genes. Furthermore, ectopic overexpression of either *htl* or *fz* in the mesoderm partially rescues the defective CM specification phenotype in embryos lacking both Forkhead genes. Together, these data emphasize the functional redundancy that leads to robustness in the cardiac progenitor specification process, and illustrate the pleiotropic functions of Forkhead TFs in different aspects of cardiogenesis.

## Abstract (precis)

**Summary:** Checkpoint suppressor homologue and Jumeau, which are known to govern cardiac progenitor cell divisions, play additional, mutually redundant roles in specifying cardiac mesoderm in *Drosophila*.

## INTRODUCTION

The rhythmically contracting heart of *Drosophila* exhibits remarkable similarities to the vertebrate heart at the primitive linear tube stage of development in terms of structure, morphogenetic origins and regulatory mechanisms (Bodmer and Frasch, 2010; Cripps and Olson, 2002; Olson, 2006). In both *Drosophila* and vertebrates, the heart tube originates from two bilaterally symmetrical rows of mesodermal cells that have migrated most distally from the point of invagination during gastrulation. This migration ensures that these cells end up in stereotyped locations where, in response to appropriate position-specific inductive signals such as bone morphogenetic proteins, Wnt proteins and fibroblast growth factors, they initiate gene expression programs involving numerous conserved transcription factors (e.g. GATA, FOG, Forkhead domain, NK homeodomain, LIM homeodomain and T-box proteins) and become determined as the cardiac mesoderm (CM), i.e. the cardiac progenitors that are the precursors of the embryonic heart. Subsequent refinement and modulation of these gene expression programs bring about the division and differentiation of these CM cells into distinct cardiac subtypes, such as the inner tube of *Myocyte enhancer factor 2* (*Mef2*)-expressing contractile cardial cells (CCs) and the external sheath of *Zn finger homeodomain 1* (*zfh1*)-expressing nephrocytic pericardial cells (PCs) in *Drosophila*.

Cardiogenesis thus requires the integration of multiple signaling pathways and transcription factor-mediated gene expression programs to orchestrate diverse developmental processes. This raises two intriguing questions: how are the numerous complex processes involved in cardiogenesis orchestrated by a finite set of regulators, and how is the requisite coordination between these distinct regulatory mechanisms achieved?

One family of transcription factors (TFs) that has been implicated in cardiogenesis in both vertebrates and *Drosophila* is the Forkhead (Fkh/Fox) domain family of proteins. At least four Fkh TFs are known to be required for proper cardiac development in mammals, and mutations in three Fkh genes have been linked to human congenital heart defects (Evans-Anderson et al., 2008; Hu et al., 2004; Korver et al., 1998; Roessler et al., 2008; Wang et al., 2004; Yu et al., 2010). We have previously also shown a cardiogenic role for two *Drosophila* Fkh genes, *jumeau* (*jumu*) and *Checkpoint suppressor homologue* (*CHES-1-like*). Both genes are initially maternally expressed, with *jumu* and *CHES-1-like* showing subsequent zygotic expression in the cells fated to become the CM from embryonic Stages 11 to 13, and from Stages 11 to 12, respectively. Each of these two Fkh genes determines cardiac cell subtypes, numbers and positions by regulating a Polo kinase-dependent pathway to mediate three distinct categories of cardiac progenitor cell divisions (Ahmad et al., 2014, 2012). In addition, our prior findings revealed that Fkh TF binding sites are significantly enriched in combination with those of other known cardiogenic TFs in the enhancers of genes expressed in the heart, and that overexpression of Jumu in the mesoderm resulted in elevated expression levels of many known cardiac genes (Ahmad et al., 2014, 2012; Zhu et al., 2012). Collectively, these results suggested that these Fkh TFs mediate additional cardiogenic processes beyond solely cardiac progenitor cell divisions by regulating many downstream target genes.

Here, we show that the Fkh genes *jumu* and *CHES-1-like* also play a significant role in specifying the CM, and that this process is achieved by the Fkh TFs transcriptionally regulating Heartless (Htl), which is a fibroblast growth factor receptor (FGFR), and Frizzled (Fz), which is a receptor of the Wingless/Wnt signaling pathway.

## RESULTS

### Loss of function of both *CHES-1-like* and *jumu* results in embryos missing entire rows of heart cells in random hemisegments

**Fig. 1.**
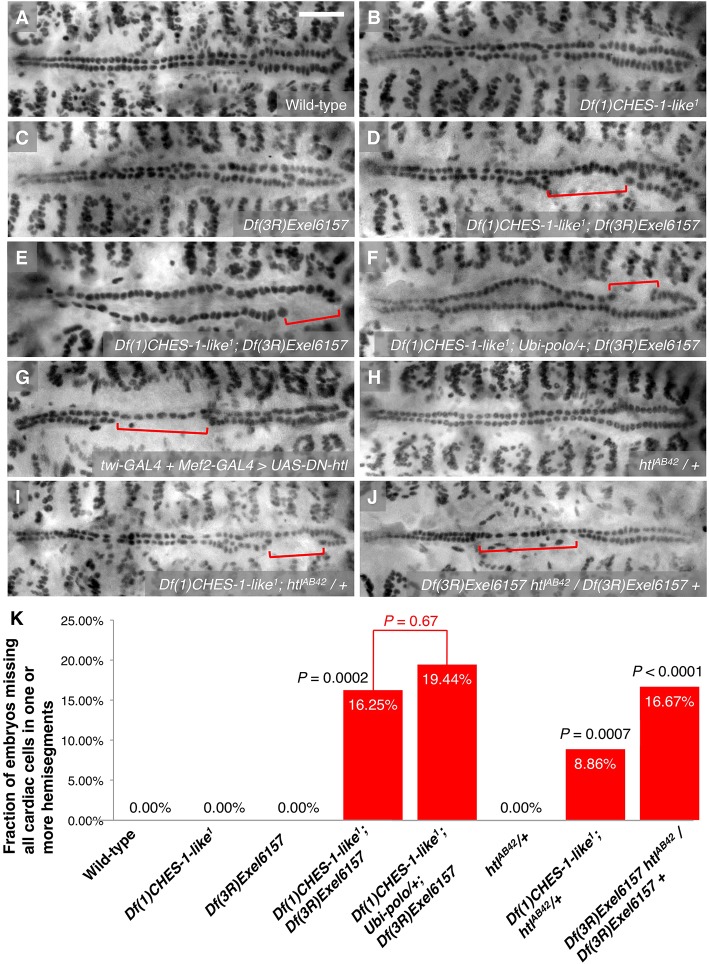
**MCH phenotypes associated with *CHES-1-like*, *jumu* and *htl*.** (A-J) Mef2 antibody staining of CCs, illustrating the presence or absence of the MCH phenotype (square brackets) in representative Stage 16 embryos that are (A) wild type, (B) homozygous for the *CHES-1-like* null mutation, (C) homozygous for the *jumu* null deficiency, (D,E) doubly homozygous for both the *CHES-1-like* mutation and the *jumu* deficiency, (F) doubly homozygous for both the *CHES-1-like* mutation and the *jumu* deficiency, but ubiquitously expressing *polo*, (G) expressing a dominant-negative version of the Htl FGFR pan-mesodermally, (H) heterozygous for a *htl* null mutation, (I) homozygous for the *CHES-1-like* null mutation and heterozygous for the *htl* null mutation, or (J) homozygous for the *jumu* null deficiency and heterozygous for the *htl* mutation. Scale bar: 50 μm. (K) Quantification and significance of the MCH phenotypes. From left to right, the *P*-values indicate the significances of the difference in phenotype between embryos lacking both Fkh genes and embryos lacking either the *CHES-1-like* or *jumu* gene; the difference in phenotype between embryos missing both Fkh genes and embryos missing both Fkh genes while ubiquitously expressing *polo*; the difference between the phenotype of embryos both heterozygous for the *htl* null mutation and homozygous for the *CHES-1-like* null mutation and the additive effects of the phenotypes in *htl* heterozygotes and the *CHES-1-like* homozygotes; and the difference between the phenotype of embryos both heterozygous for the *htl* null mutation and homozygous for the *jumu* null deficiency and the additive effects of the phenotypes in *htl* heterozygotes and the *jumu* homozygotes.

Our previous study showed that embryos homozygous for either the *jumu* null deficiency *Df(3R)Exel6157* or the *CHES-1-like* null mutation *Df(1)CHES-1-like^1^* exhibit hemisegments with localized increases or decreases in CC number, occasional enlarged CC nuclei, or mispositioned CCs as a consequence of defective cardiac progenitor cell divisions when compared with wild-type embryos (Fig. 1A-C) (Ahmad et al., 2012). However, a significantly large fraction (*P*=0.0002) of embryos lacking both *jumu* and *CHES-1-like* functions exhibits a more severe phenotype that never occurs in embryos missing just one of these two Fkh genes: 16.25% of embryos that were doubly homozygous for both the *jumu* null deficiency and the *CHES-1-like* null mutation exhibit one or more hemisegments missing entire rows of cardial cells (Fig. 1D,E,K; Table 1). Pericardial cells were also absent in the hemisegments missing CCs (Fig. S1). The location of the hemisegments lacking all heart cells was random: no significant difference (*P*=0.83) was detected in the frequency of this ‘missing cardiac hemisegments’ (MCH) phenotype between the anterior aorta and the wider posterior section of the heart (Table S1).

**Table 1.**
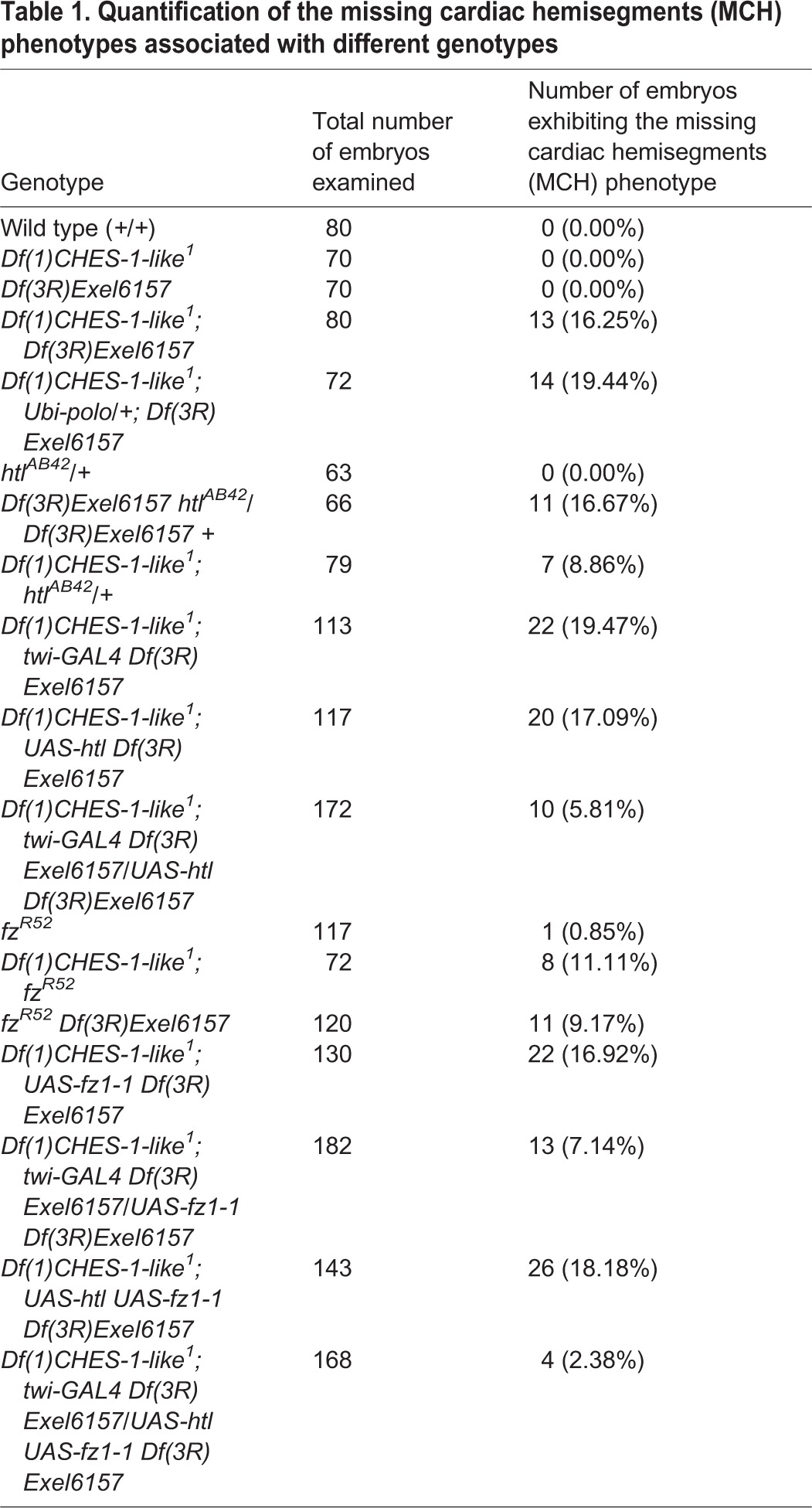
**Quantification of the missing cardiac hemisegments (MCH) phenotypes associated with different genotypes**

Similar MCH phenotypes were also observed when both *jumu* and *CHES-1-like* functions were simultaneously knocked down by CM precursor-targeted RNA interference directed by the *tinD-GAL4* and *Hand-GAL4* drivers (Fig. S2), indicating that the requirement of both these Fkh genes for proper heart development is autonomous to the cells fated to become cardiac progenitors.

This MCH phenotype could not be a consequence of the cardiac progenitor cell division defects caused by mutations in *jumu* or *CHES-1-like* for at least two reasons. First, because four of the six CCs in each hemisegment, the Tinman-expressing CCs (Tin-CCs), arise by two symmetric cell divisions from two cardiac progenitor cells (Han and Bodmer, 2003; Ward and Skeath, 2000), the aforementioned cell division defects would have left at least these two progenitor cells intact in the CC row, rather than resulting in all six CCs being lost. Second, we had previously shown that the cardiac progenitor cell division defects caused by mutations in *jumu* or *CHES-1-like* could be partially rescued by ubiquitously expressing their downstream gene *polo*, with many of the rescued hearts looking the same as wild type (Ahmad et al., 2012). However, no significant rescue (*P*=0.67) of this MCH phenotype was detected when *polo* was ubiquitously expressed in embryos lacking both *jumu* and *CHES-1-like* functions: 19.44% of these embryos exhibited the phenotype (Fig. 1F,K; Table 1).

Collectively, these data suggest that the MCH phenotype is likely to be a consequence of a defect at an earlier step in cardiogenesis: either a failure of the mesodermal cells to migrate far enough dorsally to reach the locations where they could receive the position-specific signals to become the CM, or a defect in the signal transduction mechanisms activated by these position-specific cues.

### The MCH phenotype of embryos lacking both *CHES-1-like* and *jumu* functions is not caused by defects in mesoderm migration

To determine whether the MCH phenotype in the double homozygotes reflects defects in mesoderm migration, we examined and compared wild-type embryos, embryos homozygous for a mutation of *stumps*, a gene essential for proper mesoderm migration (Imam et al., 1999; Michelson et al., 1998a; Vincent et al., 1998), and embryos that were doubly homozygous for both the *CHES-1-like* and *jumu* null mutations.

**Fig. 2.**
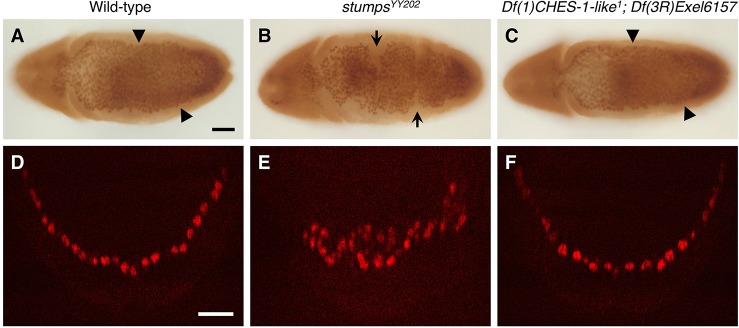
**The MCH phenotype in embryos lacking both *CHES-1-like* and *jumu* functions is not caused by defects in mesoderm migration.** (A-C) Mef2 antibody staining of the mesoderm, illustrating the presence or absence of mesoderm migration defects in ventral views of representative Stage 9 embryos that are (A) wild type, (B) homozygous for the *stumps^YY202^* mutation and (C) lack both Fkh genes. Note that the dorsolateral margin of the mesoderm in the *stumps* mutant (B) is ragged and exhibits discontinuities (arrows) characteristic of migration defects. By contrast, the migrating mesoderm of both wild-type embryos (A) and embryos lacking both *CHES-1-like* and *jumu* (C) exhibit smooth dorsolateral margins (arrowheads). Scale bar: 50 μm. (D-F) Mef2 antibody staining (red) of the migrating mesoderm in representative transverse views of the ventral halves of Stage 10 embryos that are (D) wild type, (E) lack *stumps* function, and (F) lack both Fkh genes. Note that the mesoderm migrates dorsolaterally as a monolayer in both the wild-type embryo and the *CHES-1-like; jumu* double homozygote, but remains aggregated ventrally and unable to reach the dorsalmost positions in the *stumps* mutant. Scale bar: 25 μm.

Ventral views of the migrating mesoderm in wild-type embryos reveal a smooth dorsolateral margin at Stage 9 (Fig. 2A). Transverse views show the mesoderm migrating smoothly as a monolayer with the dorsalmost mesodermal cells reaching locations by Stage 10 (Fig. 2D) where they can receive position-specific signals to become specified as cardiac progenitors. By contrast, the mesoderm of the *stumps* mutants display a ragged margin characteristic of defects in migration at Stage 9 (Fig. 2B), with transverse section views at Stage 10 showing mesodermal cells aggregated ventrally and not reaching the dorsalmost positions reached by cells in wild-type embryos (Fig. 2E).

None of the 110 Stage 9 doubly homozygous *CHES-1-like; jumu* embryos we examined displayed the ragged mesodermal margin characteristic of migration defects (Fig. 2C). Furthermore, examination of 20 Stage 10 transverse views of these double homozygotes showed that mesodermal cells always migrated as a monolayer and were always able to reach the dorsalmost positions necessary for receiving instructive cardiogenic signals (Fig. 2F). Collectively, these results indicate that migration defects are not responsible for this MCH phenotype.

### Loss of the late function of the FGFR Heartless phenocopies the MCH phenotype of embryos lacking both *CHES-1-like* and *jumu* functions

As the MCH phenotype was not due to defects in mesoderm migration, we considered the alternative possibility that this phenotype reflected defects in signaling mechanisms involved in cardiac progenitor specification. A previously known example of such CM specification defects involves disruption of signal transduction through the FGFR Heartless (Htl) (Michelson et al., 1998b). Although *htl* initially plays a role in mesoderm migration, it also has a second, later function in specifying the CM. When this late function of *htl* is disrupted by expressing a dominant-negative version of the FGFR throughout the entire mesoderm such that migration is not affected, MCH phenotypes identical to those in the *CHES-1-like*; *jumu* double mutants are observed (Fig. 1G).

### Synergistic genetic interactions between the Fkh genes and *htl*

The observation that disrupting late *htl* function phenocopies the MCH phenotype of *CHES-1-like; jumu* double mutants raised the possibility that *htl* and the Fkh genes could be acting together to specify the CM. In such a case, strong genetic interactions might occur between either of the Fkh genes and *htl*. To examine this possibility, we quantified and compared the MCH phenotypes in embryos heterozygous for a *htl* null mutation, in embryos homozygous for a *CHES-1-like* null mutation, in embryos homozyogous for a *jumu* null deficiency, in embryos both heterozygous for the *htl* mutation and homozygous for the *CHES-1-like* mutation, and in embryos heterozygous for the *htl* allele and homozygous for the *jumu* deficiency (Fig. 1K; Table 1). Whereas the MCH phenotype was never detected in the *htl* heterozygotes (Fig. 1H) or in the *CHES-1-like* homozygotes (Fig. 1B), embryos that were both heterozygous for the *htl* allele and homozygous for the *CHES-1-like* allele (Fig. 1I) exhibited this phenotype with a frequency that was significantly greater (*P*=0.0007) than the additive effects of the phenotypes in *htl* heterozygotes and the *CHES-1-like* homozygotes (Fig. 1K). Similarly, the frequency of MCH phenotypes in embryos both heterozygous for the *htl* mutation and homozygous for the *jumu* deficiency (Fig. 1J) was significantly greater (*P*<0.0001) than the additive effects of the *htl* heterozygotes and *jumu* homozygotes (Fig. 1C,H,K). Collectively, these results demonstrate synergistic genetic interactions between *CHES-1-like* and *htl*, and between *jumu* and *htl*, and are consistent with all three genes acting in the same CM specification pathway.

### *CHES-1-like* and *jumu* transcriptionally activate *htl* expression in the precursors of the cardiac mesoderm

Although the synergistic genetic interactions between *CHES-1-like*, *jumu* and *htl* suggests that they work together in the same cardiac progenitor specification pathway, they do not indicate whether the Fkh TFs act either exclusively or redundantly upstream of *htl* to regulate its expression, or whether signaling through Htl instead mediates the expression of the Fkh genes. To discriminate between these possibilities, we examined the expression of *htl* in embryos that were wild type, embryos that were mutant for one or the other of the two Fkh genes, and embryos that lacked both Fkh genes.

**Fig. 3.**
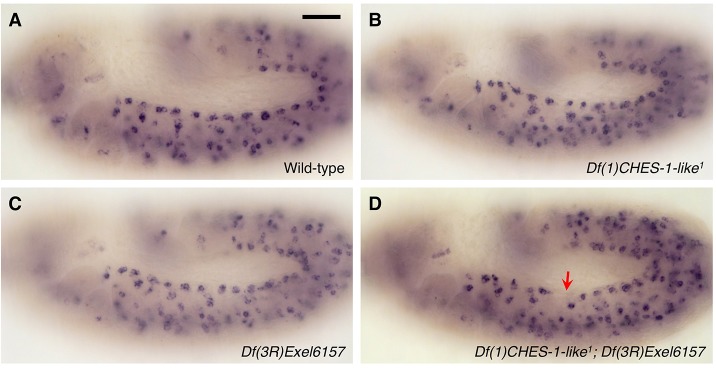
***CHES-1-like* and *jumu* activate *htl* transcription in the cardiac mesoderm.** (A-D) *htl* mRNA in Stage 11 wild-type embryos (A), embryos lacking *CHES-1-like* function (B), embryos lacking *jumu* function (C), and embryos lacking both *CHES-1-like* and *jumu* functions (D) detected by *in situ* hybridization. The arrow in D indicates the absence of *htl* expression in cells that would normally have become the CM. Scale bar: 50 μm.

Whole embryo *in situ* hybridizations showed that *htl* transcripts are expressed in an evenly distributed pattern in every hemisegment in wild-type embryos when the CM is specified at Stage 11 (Fig. 3A). Although there was no significant change in *htl* expression in embryos homozygous for mutations in either *CHES-1-like* or *jumu* alone (Fig. 3B,C), hemisegments lacking *htl* expression in cells that would normally have become the CM were frequently observed in embryos lacking both *CHES-1-like* and *jumu* functions (Fig. 3D). These results are consistent with both Fkh genes acting in a mutually redundant manner with either one being sufficient to activate *htl* expression.

### *CHES-1-like* and *jumu* transcriptionally activate *htl* expression by binding to the cardiac enhancer ChIPCRM2610

**Fig. 4.**
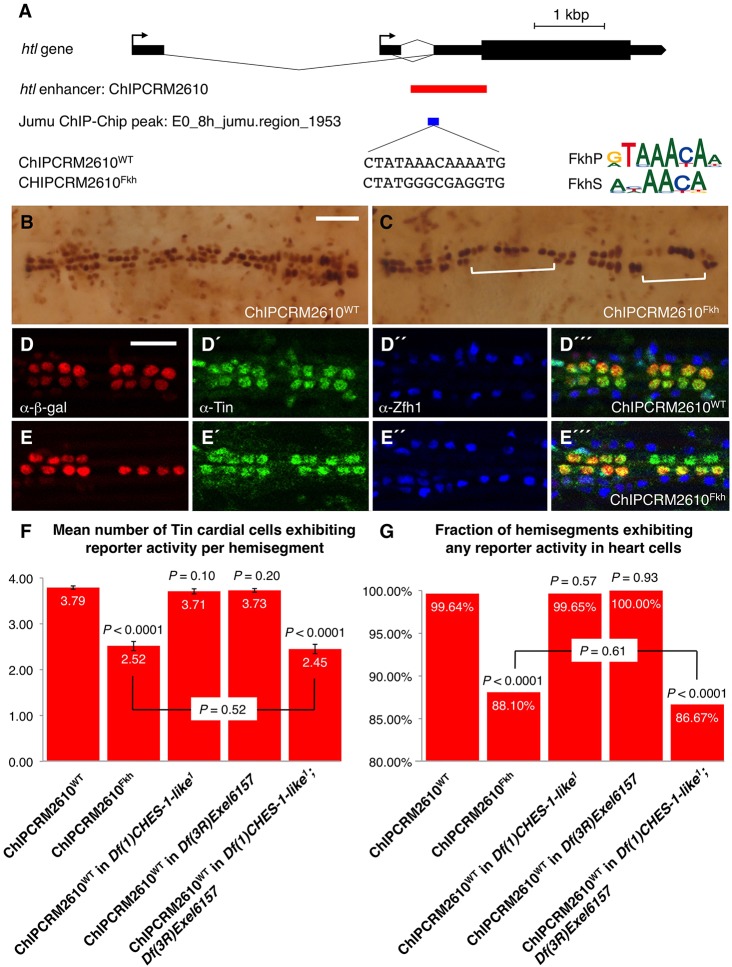
**CHES-1-like and Jumu are activators of a *htl* cardiac enhancer.** (A) Relative positions of the *htl* gene, the *htl* enhancer ChIPCRM2610, the Jumu ChIP peak, and the Fkh TF binding sequence. Logo representations of the PWMs of Fkh primary (FkhP) and secondary (FkhS) TF binding motifs obtained from protein-binding microarrays are shown, as are the Fkh binding sequences in the wild-type (ChIPCRM2610^WT^) and the mutated (ChIPCRM2610^Fkh^) enhancer. (B,C) Representative Stage 16 hearts showing β-galactosidase reporter activity driven by the wild-type (B) and mutant (C) enhancers. Square brackets indicate entire hemisegments lacking reporter activity in the case of the mutated enhancer (C). Scale bar: 50 μm. (D-E‴) Enhancer activity at single-cell resolution. The posterior-most four CCs in a hemisegment are marked by Tin expression (green), and the PCs are marked by Zfh1 expression (blue). (D-D‴) Reporter activity (red) driven by the wild-type enhancer is located primarily in the Tin-expressing CCs. (E-E‴) Mutating the Fkh binding site results in significant reduction of reporter activity. Scale bar: 25 μm. (F,G) Quantification and significance of the effects of mutating the Fkh binding site in the ChIPCRM2610 *htl* enhancer, and the effects of loss of function of *CHES-1-like*, *jumu*, or both *CHES-1-like* and *jumu* on the wild-type ChIPCRM2610 *htl* enhancer activity. The *P*-value over each column indicates the significance of the difference in reporter activity between the relevant enhancer genotype combination and the wild-type ChIPCRM2610^WT^ enhancer in wild-type embryos. Error bars in F indicate 95% confidence intervals.

We next attempted to ascertain whether this Fkh-mediated transcriptional regulation was achieved by direct binding of the CHES-1-like and Jumu TFs to an *htl* enhancer. A particularly promising candidate for the *htl* enhancer through which this Fkh-mediated activation might be brought about is ChIPCRM2610, which drives reporter expression in the CM (Gisselbrecht et al., 2013). Chromatin immunoprecipitation data from the modENCODE project (Nègre et al., 2011) indicated that Jumu protein binds *in vivo* to ChIPCRM2610 at the CM specification stage (Fig. 4A). Utilizing known binding specificities of mouse Fkh TFs (Badis et al., 2009; Robasky and Bulyk, 2011) that we had previously used successfully to analyze Fkh function in *Drosophila* enhancers (Zhu et al., 2012), we identified a single putative Fkh binding site in ChIPCRM2610 the sequence of which matched those of both the canonical primary Fkh binding motif (FkhP) and a secondary alternative Fkh binding motif (FkhS), and the location of which corresponded with the Jumu chromatin immunoprecipitation data (Fig. 4A).

To test whether the Fkh TFs regulate ChIPCRM2610 through this single putative Fkh binding site, we used relevant protein binding microarray data to mutate the wild-type Fkh binding site to a sequence that, based on prior findings (Robasky and Bulyk, 2011; Zhu et al., 2012), results in significant loss of Fkh TF binding (Fig. 4A). The wild-type and mutant versions of the ChIPCRM2610 *htl* enhancer were cloned into β-galactosidase reporter vectors and independently inserted into the same location on the second chromosome. Thus, any differences in reporter expression between the wild-type and mutant *htl* enhancers can be attributed solely to the Fkh binding site mutation and not to local positional effects.

As the *Drosophila* heart at embryonic Stage 16 consists of multiple cells arranged in a metamerically repeated and stereotyped pattern, the perdurance of the expression patterns of the wild-type and mutant ChIPCRM2610 enhancer in each repeated hemisegment at this stage provides a simple means of scoring enhancer activity. The wild-type enhancer (ChIPCRM2610^WT^) drives reporter expression in virtually every cardiac hemisegment (99.64% of all examined hemisegments) primarily in the four Tin-CCs (Fig. 4B,D-D‴,F,G; Table 2; Fig. S3A). By contrast, mutating the Fkh binding site (resulting in the ChIPCRM2610^Fkh^ enhancer) causes significant reduction (*P*<0.0001) of enhancer-driven reporter expression, with many hemisegments (11.90%) showing no cardiac reporter expression at all, and an overall decrease in the mean number of Tin-CCs per hemisegment in which the reporter is expressed from 3.79 to 2.52 (Fig. 4C,E-E‴,F,G; Table 2; Fig. S3B).

**Table 2.**
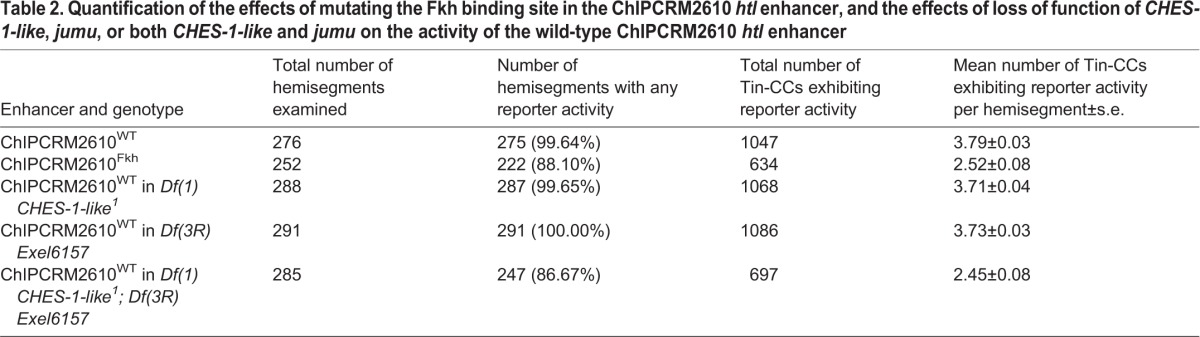
**Quantification of the effects of mutating the Fkh binding site in the ChIPCRM2610** ***htl*** **enhancer, and the effects of loss of function of** ***CHES-1-like***, ***jumu***, **or both** ***CHES-1-like*** **and** ***jumu*** **on the activity of the wild-type ChIPCRM2610** ***htl*** **enhancer**

We next assessed whether a similar phenotype could be obtained by eliminating the function of one or both of the Fkh TFs in *trans*. Embryos lacking either *CHES-1-like* or *jumu* exhibited reporter expression driven by the wild-type ChIPCRM2610^WT^ enhancer that was not significantly different (*P*=0.10 and *P*=0.20, respectively, for mean numbers of Tin-CCs per hemisegment exhibiting reporter expression; *P*=0.57 and *P*=0.93, respectively, for the fractions of all hemisegments exhibiting any reporter expression) from that in wild-type embryos (Fig. 4F,G; Fig. S3C-D‴; Table 2).

By contrast, embryos that lacked both Fkh TFs demonstrated a significant reduction (*P*<0.0001) in the number of cells with visible reporter expression, with expression being limited to a mean of 2.45 Tin-CCs per hemisegment, and 13.33% of the hemisegments examined showing no cardiac expression (Fig. 4F,G; Fig. S3E-E‴; Table 2). Of note, there was no significant difference in reporter expression (*P*=0.52 for mean numbers of Tin-CCs exhibiting reporter expression; *P*=0.61 for fractions of all hemisegments exhibiting any reporter expression) between these double homozygotes bearing the ChIPCRM2610^WT^ enhancer and otherwise wild-type embryos bearing the ChIPCRM2610^Fkh^ enhancer (Fig. 4F,G). These observations indicate that abolition of Fkh TF binding by either eliminating both CHES-1-like and Jumu TFs, or by mutating the binding site on the enhancer results in similar levels of reduction in enhancer activity.

Collectively, the convergence of results between these *cis* and *trans* experiments demonstrate (1) that the CHES-1-like and Jumu TFs activate *htl* expression in the precursors of the CM by directly binding to the ChIPCRM2610 enhancer, and (2) that either of these two Fkh TFs is sufficient by itself to activate expression in wild-type numbers of cells.

### Mesoderm-targeted ectopic expression of Htl partially rescues the MCH phenotype in embryos lacking both *CHES-1-like* and *jumu* functions

**Fig. 5.**
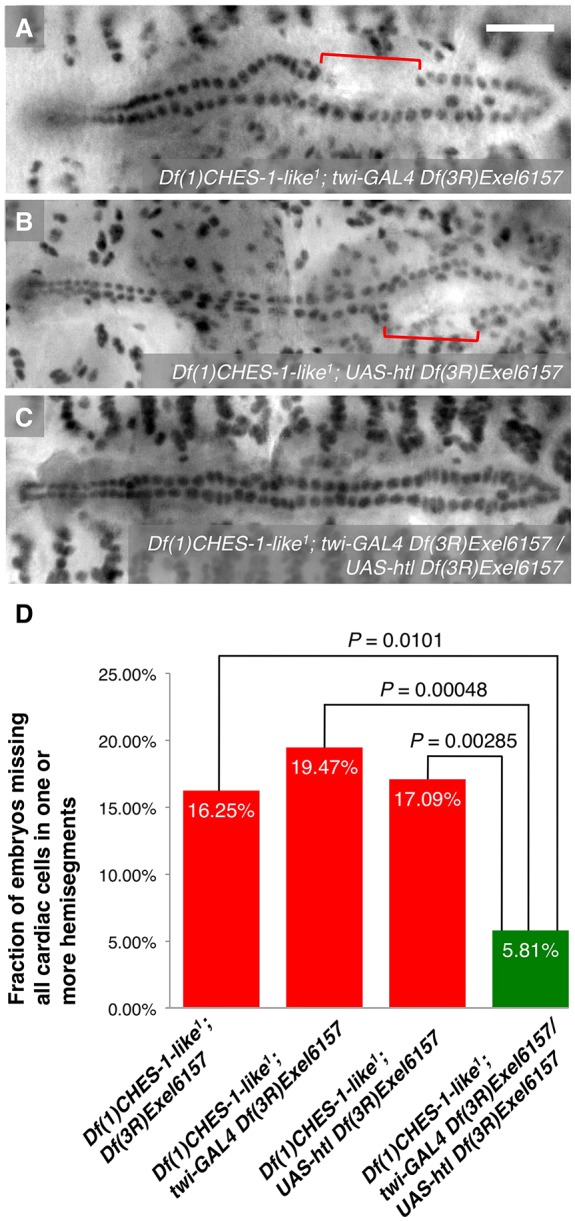
**Mesoderm-targeted ectopic expression of Htl partially rescues the MCH phenotype in embryos lacking both *CHES-1-like* and *jumu* functions.** (A-C) Mef2 antibody staining of CCs, illustrating the presence or absence of the MCH phenotype (square brackets), in representative Stage 16 embryos lacking both functional *CHES-1-like* and *jumu* genes but possessing (A) *twi-GAL4*, (B) *UAS-htl* or (C) both *twi-GAL4* and *UAS-htl* transgenes, and therefore expressing Htl constitutively throughout the entire mesoderm. Scale bar: 50 μm. (D) Quantification and significance of the MCH phenotypes.

The results from our genetic interaction experiments together with our *cis* and *trans* analysis of *htl* expression suggest that *CHES-1-like* and *jumu* transcriptionally activate *htl* expression to bring about proper CM specification. To test this hypothesis, full length Htl (Michelson et al., 1998b) was expressed pan-mesodermally under the control of the *twi-GAL4* driver. If *htl* acts downstream of the Fkh TFs in this CM specification pathway, then ectopic pan-mesodermal expression of the FGFR should at least be able to partially rescue the MCH phenotype of the *CHES-1-like; jumu* double mutants. We found that expression of the FGFR throughout the mesoderm does indeed significantly reduce the severity of the MCH phenotype in embryos lacking both *CHES-1-like* and *jumu* functions (Fig. 5; Table 1), indicating a downstream requirement of *htl* for correct CM specification mediated by the Fkh genes.

### Microarray-based genome-wide RNA expression profiling indicates that *CHES-1-like* and *jumu* differentially regulate the expression of many known cardiac genes

Our previous findings had shown that Fkh TF binding sites are significantly enriched in combination with those of other known cardiogenic TFs in the enhancers of genes expressed in the CM or heart (Ahmad et al., 2014; Zhu et al., 2012), suggesting that these Fkh TFs regulate numerous downstream cardiac genes. To test this hypothesis, we used flow cytometry and Affymetrix microarrays to examine the effects of homozygous null mutations for either *CHES-1-like* or *jumu* on gene expression in mesodermal cells from Stage 11 embryos.

Consistent with the observation that *CHES-1-like* and *jumu* both activate cardiac genes, such as *htl*, and repress others, such as *Nidogen* (Zhu et al., 2012), our genome-wide expression profiling identified 42 and 56 known heart genes that were downregulated and upregulated, respectively, in the mesoderm of *CHES-1-like* null mutants, and 38 and 39 known cardiac genes that were downregulated and upregulated, respectively, in *jumu* null mutants (Table S2A-D). These numbers represent statistically significant over-representations (*P*=1.907×10^−11^ for *CHES-1-like*; *P*=6.987×10^−9^ for *jumu*) of known cardiac genes among those for which expression is differentially regulated by the Fkh genes, thus confirming that the Fkh TFs do indeed regulate many heart genes.

Of note, significant reduction in the expression levels of *htl* is not detected in the microarray data for either *CHES-1-like* or *jumu* null mutants alone (Table S2A,C). The most conservative explanation for this observation is that it reflects the functional redundancy already demonstrated between CHES-1-like and Jumu in regulating *htl* expression: either of these two Fkh TFs is sufficient by itself to activate wild-type levels of *htl* expression. Consistent with this explanation, microarray-based genome-wide expression profiling data from mesodermal cells ectopically overexpressing *jumu* (Ahmad et al., 2012) showed elevated expression levels of *htl* (log_2_ fold change=0.990804; *P*=8.97×10^−5^) compared with wild type (Table S2F).

If *jumu* is indeed sufficient to activate *htl*, then ectopic overexpression of Jumu in the mesoderm would be expected to partially resemble the effects of ectopic overexpression of activated Htl. This result is indeed observed in our expression profiling data: there is significant overlap (odds ratio=4.566; *P*<8.8×10^−16^) between genes with significantly elevated expression levels as a consequence of ectopic mesodermal overexpression of the constitutively activated form of Htl (Ahmad et al., 2012; Estrada et al., 2006) and genes that are upregulated when *jumu* is ectopically overexpressed in the mesoderm (Table S2F,G).

### Expression profiling indicates that *CHES-1-like* and *jumu* also activate mesodermal expression of the Wingless/Wnt receptor-encoding gene *frizzled*

CM specification in both *Drosophila* and vertebrates also involves other signaling mechanisms besides FGF/FGFR, such as the Wingless/Wnt, Bone morphogenetic protein and Notch pathways (Bodmer and Frasch, 2010; Vincent and Buckingham, 2010). This raises the question of whether components of these other signaling pathways involved in cardiac progenitor specification are also regulated by the Fkh TFs. To address this, we again examined the expression profiling data for *CHES-1-like* and *jumu* null mutants.

We found that *frizzled* (*fz*), which encodes a receptor of the Wnt signaling protein Wingless (Wg), had its expression levels significantly reduced in the mesoderm of embryos lacking either *CHES-1-like* (log_2_ fold change=−1.257736; *P*=3.16×10^−7^) or *jumu* function (log_2_ fold change=−1.2118035; *P*=2.59×10^−7^) compared with wild type (Table S2A,C). These results indicate that both *jumu* and *CHES-1-like* activate *fz* expression in the mesoderm.

As Fz is one of the receptors of Wg, reductions in the expression levels of *fz* in the mesoderm would be expected to partially resemble the effects of inhibiting or reducing Wg signaling, such as with null mutations in *wg*. Expression profiling data on flow cytometry-purified mesodermal cells show that there is indeed significant overlap between genes downregulated in the mesoderm of embryos homozygous for a null mutation in *wg* (Ahmad et al., 2012; Estrada et al., 2006) and genes downregulated in either *CHES-1-like* (odds ratio=2.131; *P*<8.8×10^−16^) or *jumu* (odds ratio=1.786; *P*<8.8×10^−16^) null mutants, thus providing further evidence for the Fkh TF-mediated activation of *fz* (Table S2A,C,E).

### Synergistic genetic interactions between the Fkh genes and *fz*

The largely ubiquitous presence of the *fz* transcript at Stages 10-11 due to maternal expression (Park et al., 1994) made it impossible for us to independently verify its Fkh-mediated activation in the subset of mesodermal cells normally destined to become cardiac progenitors by performing RNA *in situ* hybridization in appropriate wild-type and Fkh mutant backgrounds. The absence of known cardiac-specific enhancers for *fz* also precluded us from carrying out *cis* and *trans* analyses as in the case of *htl*. However, we hypothesized that if one or both of the Fkh genes were indeed regulating *fz* expression in the CM specification process, then we might expect to see synergistic, i.e. more than merely additive, genetic interactions between the Fkh TFs and the Wg receptor.

**Fig. 6.**
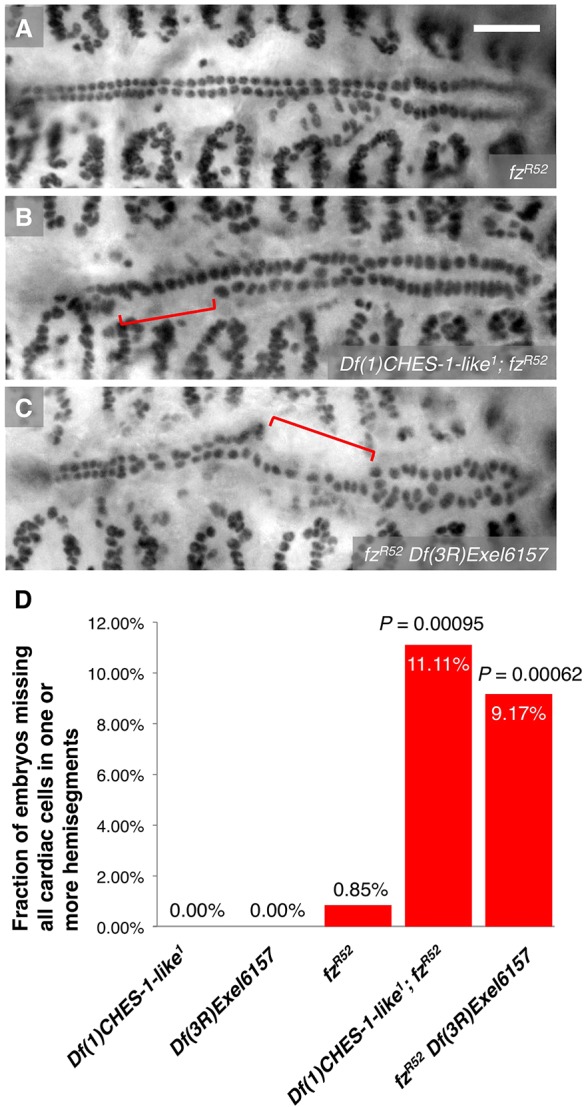
**Synergistic genetic interactions between *CHES-1-like* and *fz*, and between *jumu* and *fz*.** (A-C) Mef2 antibody staining of CCs, illustrating the presence or absence of the MCH phenotype (square brackets), in representative Stage 16 embryos that are (A) homozygous for *fz^R52^*, a strong hypomorphic mutation of *fz*, (B) doubly homozygous for both the *fz* mutation and the *CHES-1-like* null mutation, and (C) doubly homozygous for both the *fz* mutation and the *jumu* null deficiency. Whereas the MCH phenotype is rarely detected in the *fz^R52^* homozygotes, embryos doubly homozygous for the *fz* mutation and one of the Fkh gene mutations exhibit significant instances of the MCH phenotype. Scale bar: 50 μm. (D) Quantification and significance of the MCH phenotypes. From left to right, the *P*-values indicate the difference between the phenotype of embryos doubly homozygous for the *fz* and *CHES-1-like* mutations and the additive effects of the phenotypes in *fz* homozygotes and *CHES-1-like* homozygotes; and the difference between the phenotype of embryos doubly homozygous for the *fz* mutation and the *jumu* null deficiency and the additive effects of the phenotypes in *fz* homozygotes and *jumu* homozygotes.

Previous work had already identified a role for *fz* in CM specification: embryos lacking both Fz and another Wg receptor, Frizzled 2 (Fz2), fail to develop cardiac progenitors (Bhanot et al., 1999; Chen and Struhl, 1999). Given this redundancy between the two Wg receptors and the maternal expression of *fz*, when we examined Stage 16 embryos homozygous for *fz^R52^*, a strong hypomorphic allele of *fz* resulting in a truncated protein (Jones et al., 1996), we found that only one out of 117 homozygotes (0.85%) exhibited the MCH phenotype (Fig. 6A,D; Table 1).

Embryos individually homozygous for either the *CHES-1-like* null mutation (Fig. 1B) or the *jumu* null deficiency (Fig. 1C) do not exhibit the MCH phenotype (Fig. 6D; Table 1). However, embryos that were doubly homozygous for mutations in both *CHES-1-like* and *fz* exhibited the MCH phenotype with a frequency (11.11%) significantly greater (*P*=0.00095) than the additive effects of the phenotypes in *CHES-1-like* homozygotes and the *fz* homozygotes (Fig. 6B,D; Table 1). Similarly, the frequency of MCH phenotypes (9.17%) in embryos doubly homozygous for both the *fz* hypomorphic mutation and the *jumu* null deficiency was significantly greater (*P*=0.00062) than the additive effects of the single *fz* and *jumu* homozygotes (Fig. 6C,D; Table 1).

Collectively, these results demonstrate synergistic genetic interactions between *CHES-1-like* and *fz*, and between *jumu* and *fz*. In light of our expression profiling results showing regulation of *fz* in the mesoderm by both *CHES-1-like* and *jumu*, the most conservative explanation for these synergistic genetic interactions is that both Fkh genes activate *fz* expression to bring about the proper specification of cardiac progenitors. Although these data cannot completely rule out the possibility that the Fkh genes and *fz* specify cardiac CM by independent parallel pathways, we note that in the latter scenario the genetic interactions between the Fkh genes and *fz* would be far more likely to be additive, rather than synergistic.

### Mesoderm-targeted ectopic expression of Fz partially rescues the MCH phenotype in embryos lacking both *CHES-1-like* and *jumu* functions

**Fig. 7.**
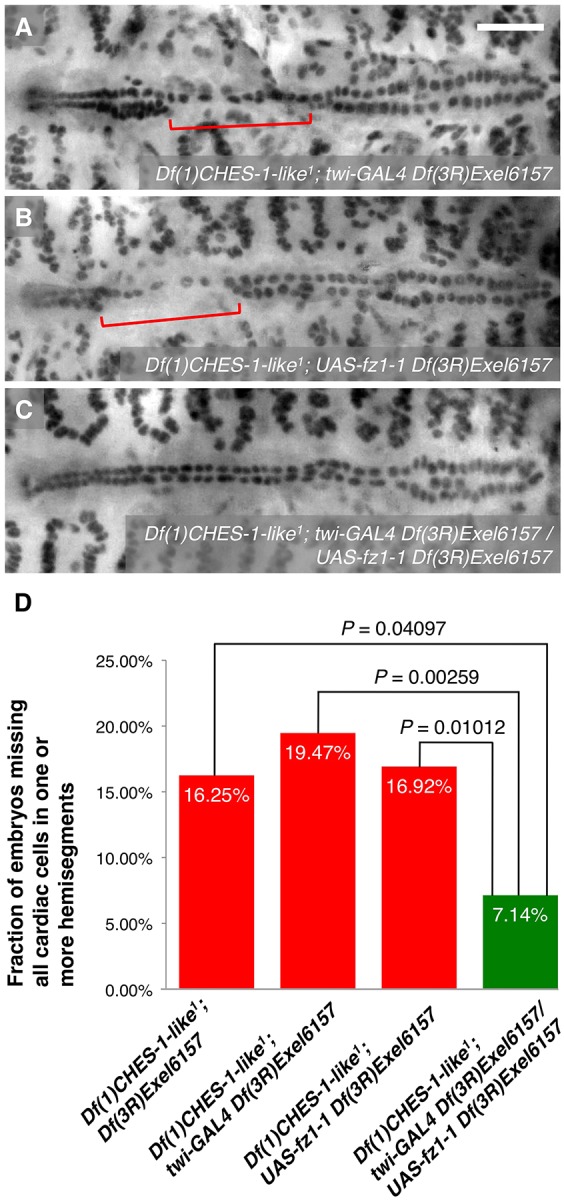
**Mesoderm-targeted ectopic expression of Fz partially rescues the MCH phenotype in embryos lacking both *CHES-1-like* and *jumu* functions.** (A-C) Mef2 antibody staining of CCs, illustrating the presence or absence of the MCH phenotype (square brackets), in representative Stage 16 embryos lacking both functional *CHES-1-like* and *jumu* genes but possessing (A) *twi-GAL4*, (B) *UAS-fz1-1* or (C) both *twi-GAL4* and *UAS-fz1-1* transgenes, and therefore expressing Fz constitutively throughout the entire mesoderm. Scale bar: 50 μm. (D) Quantification and significance of the MCH phenotypes.

To determine conclusively whether *CHES-1-like* and *jumu* also activate *fz* expression to bring about the proper CM specification, full length Fz was expressed pan-mesodermally under the control of the *twi-GAL4* driver. If *fz* acts downstream of the Fkh TFs in this CM specification pathway, then ectopic pan-mesodermal expression of Fz should be able to partially rescue the MCH phenotype of the *CHES-1-like; jumu* double mutants in a manner similar to that of ectopic pan-mesodermal expression of Htl. We found that expression of Fz throughout the mesoderm significantly reduces the severity of the MCH phenotype in embryos lacking both *CHES-1-like* and *jumu* functions (Fig. 7; Table 1), thereby confirming that the Fkh genes do indeed activate *fz* expression to bring about the proper specification of cardiac progenitors.

### Simultaneous mesoderm-targeted ectopic expression of both Htl and Fz reduces the severity of the MCH phenotype in embryos lacking both *CHES-1-like* and *jumu* functions to wild-type levels

**Fig. 8.**
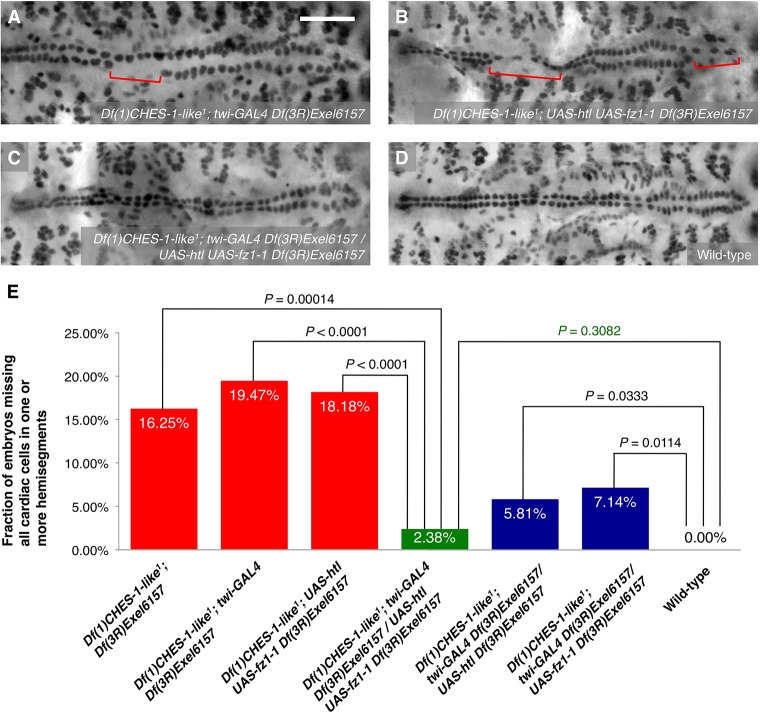
**Simultaneous mesoderm-targeted ectopic expression of both Htl and Fz reduces the severity of the MCH phenotype in embryos lacking both *CHES-1-like* and *jumu* functions to wild-type levels.** (A-C) Mef2 antibody staining of CCs, illustrating the presence or absence of the MCH phenotype (square brackets), in representative Stage 16 embryos lacking both functional *CHES-1-like* and *jumu* genes but possessing (A) *twi-GAL4*, (B) *UAS-htl* and *UAS-fz1-1*, or (C) *twi-GAL4*, *UAS-htl* and *UAS-fz1-1* transgenes, and therefore simultaneously expressing Htl and Fz constitutively throughout the entire mesoderm. Scale bar: 50 μm. (D) Mef2 antibody staining of CCs in a representative Stage 16 wild-type embryo. (E) Quantification and significance of the MCH phenotypes.

Finally, given that ectopic pan-mesodermal expression of either Htl or Fz receptors individually results in partial rescue of the MCH phenotype, we examined the effects of simultaneously expressing both these full-length proteins in embryos lacking both Fkh genes. Expressing both receptors under the control of the *twi-GAL4* driver significantly reduces the severity of the MCH phenotype in the Fkh double mutants to a level that is even lower than those obtained when either Htl or Fz are ectopically expressed individually (Fig. 8; Table 1). Of note, although significant rescue of the MCH phenotype was indeed observed with ectopic mesoderm-targeted expression of either receptor alone (Figs 5, 7), the severity of the phenotype in each case was significantly greater than that in wild-type embryos (Fig. 8E). By contrast, expressing both Htl and Fz simultaneously in the *CHES-1-like; jumu* double mutants reduces the occurrence of the MCH phenotype to levels that are not significantly different from that in wild-type embryos (Fig. 8E; Table 1). Collectively, these results indicate that the two Fkh TFs, CHES-1-like and Jumu, mediate CM specification by activating the expression of receptors of both the FGF and Wg signaling pathways.

## DISCUSSION

In this study, we demonstrate that the two *Drosophila* Fkh TFs CHES-1-like and Jumu function in a redundant manner to regulate cardiac progenitor specification, with each TF being sufficient to ensure proper specification of the CM, and the MCH phenotype being detected only when the functions of both Fkh genes are eliminated. Utilizing *in vivo* ChiP binding data, *cis*-*trans* enhancer analyses, examination of transcript expression by *in situ* hybridization in appropriate mutants, phenotypic analyses of these same mutants, and genetic interaction and rescue assays, we show that one of the pathways by which this CM specification process is achieved involves the transcriptional activation of the FGFR-encoding gene *htl* by the Fkh TFs binding to its enhancer. Our expression profiling results combined with genetic interaction and rescue assays further indicate that the same Fkh TFs mediate an additional pathway involved in this process of cardiac progenitor specification by also activating the expression of the Wg/Wnt receptor encoding gene *fz*.

### Regulation of cardiac progenitor specification by control of the expression of receptors of relevant signaling pathways

Studies of the signaling pathways involved in cardiac mesoderm/cardiac progenitor specification have focused largely on the signal transduction mechanisms themselves or on the mechanisms responsible for appropriate expression of the relevant signaling ligands. Our study highlights yet another mechanism by which CM specification is modulated: regulating the expression of the receptors involved in these signaling pathways by the activity of upstream TFs. Particularly in the case of the FGFR Htl, we found that the Fkh TFs transcriptionally activate its requisite expression in a tight spatiotemporal domain that corresponds to the cells destined to become cardiac progenitors in the dorsal mesoderm, independent of any effect of Htl on cell migration (Beiman et al., 1996; Gisselbrecht et al., 1996; Shishido et al., 1997).

The instructive role of FGF/FGFR signaling in specifying cardiac progenitors has been shown in mouse, chick and zebrafish (Alsan and Schultheiss, 2002; Barron et al., 2000; Crossley and Martin, 1995; Reifers et al., 2000) in addition to *Drosophila*. Fkh TF-mediated regulation of FGFRs in non-cardiogenic processes has also been observed in vertebrates (Coffer and Burgering, 2004; Mandinova et al., 2009; Nakada et al., 2009; Tsai et al., 2003). Given the conservation of both genes and regulatory networks in heart development in both *Drosophila* and vertebrates (Cripps and Olson, 2002; Olson, 2006), and the numerous cardiogenic processes in mammals in which the Fkh genes are involved (Evans-Anderson et al., 2008; Hu et al., 2004; Korver et al., 1998; Roessler et al., 2008; Wang et al., 2004; Yu et al., 2010), it will be of particular interest to see if cardiac progenitor specification in vertebrates also involves the regulation of relevant FGFRs by Fkh genes.

Although signaling through the Wg pathway in *Drosophila* is essential for normal heart formation, the role of Wnt signaling in specifying cardiac progenitors in vertebrates is considerably more complex: signaling through Wnt3a is required for initial mesoderm formation but inhibits later cardiac specification, whereas signaling through Wnt5a and Wnt11 stimulates cardiac progenitor specification (Gessert and Kuhl, 2010). Regardless, regulation of Fz homologs in vertebrates could provide another mechanism for modulating this process.

We had previously suggested that the Fkh gene *FoxM1*, which transcriptionally regulates a mammalian *polo* ortholog (Laoukili et al., 2005), and mutations in which exhibit cardiac progenitor cell division defects in mouse hearts (Korver et al., 1998), was the functional ortholog of *CHES-1-like* and *jumu* in mammals (Ahmad et al., 2012). Thus, it was intriguing to learn that *FoxM1* also regulates at least one mammalian *fz* homolog, Frizzled family receptor 6 (*Fzd6*) (Kim et al., 2005). Given that *FZD6* is also expressed in the human heart (Uhlen et al., 2010), it will be of interest to see if this Wnt receptor, or some other Fkh-regulated Fz homolog, also plays a role in cardiac specification.

### Redundancy and robustness in cardiac mesoderm specification

Crucial developmental steps are frequently robust and interconnected in order to prevent damage to any one of their constituent genetic components resulting in the overall disruption of the process in which they are involved. This concept is demonstrated at multiple levels by the CM specification process examined in the present study.

One elegant illustration of this regulatory design feature is the degree of mutual redundancy exhibited by *CHES-1-like* and *jumu*, with either of these genes being able to compensate for defects in the function of the other to mediate normal heart formation. That is, each Fkh TF is individually sufficient to activate the expression of *htl* to bring about proper FGFR-mediated CM specification, with the absence of either the *htl* transcript or *htl* enhancer activity in a hemisegment, as well as the presence of the MCH phenotype, being detected only when both Fkh functions are eliminated in the same embryo.

Furthermore, the absence of both Fkh TFs reduces but does not completely eliminate CM specification; similarly, the expression of the *htl* enhancer and transcript is reduced but not completely abrogated in embryos lacking both Fkh functions. These observations indicate that additional redundant mechanisms must exist to at least partially maintain *htl* expression and CM specification in the absence of the Fkh TFs. A preliminary computational analysis we performed using the algorithm PhylCRM (Warner et al., 2008) identified a highly conserved Pointed binding site in the *htl* enhancer ChIPCRM2610 as well as several putative Twist and Tinman binding sites, suggesting that these cardiogenic TFs, plus potentially other TFs not presently known to function in heart development, might mediate redundant mechanisms for activating *htl* expression and thereby CM specification in embryos lacking *CHES-1-like* and *jumu* functions.

Yet another example of an interconnection among the components of the cardiac gene regulatory network is the control of not one, but two CM specification pathways – the FGF/FGFR signaling pathway and the Wnt signaling pathway – by these Fkh TFs. Although we cannot rule out the possibility that the Fkh TFs might also regulate additional CM specification pathways, the observation that ectopic expression of both Htl and Fz simultaneously can restore wild-type levels of CM specification in the *CHES-1-like; jumu* double mutants suggests that the Fkh-mediated regulation of only these two receptors is sufficient for wild-type function.

Finally, a fourth example of the robustness of this system is the previously known redundancy in the Wnt signaling pathway itself. Fz is not the only receptor utilized by the Wnt signaling pathway for CM specification: the functions of both Fz and Fz2 have to be eliminated before any major disruption is detected in cardiac progenitor specification (Bhanot et al., 1999; Chen and Struhl, 1999).

### Pleiotropic roles of *CHES-1-like* and *jumu* in cardiogenesis

Our previous studies had suggested that the Fkh TFs might play roles in multiple cardiogenic processes. We had observed a statistically significant over-representation of primary and secondary Fkh TF binding motifs in combination with other known cardiogenic TF binding sites in putative enhancers of genes known to be expressed in the heart (Zhu et al., 2012). Machine-learning modeling of cardiac enhancers based on sequence features of known heart enhancers had also identified Fkh TFs as important regulators (Ahmad et al., 2014). Furthermore, overexpression of Jumu in the mesoderm resulted in elevated expression levels of many known cardiac genes (Ahmad et al., 2012).

**Fig. 9.**
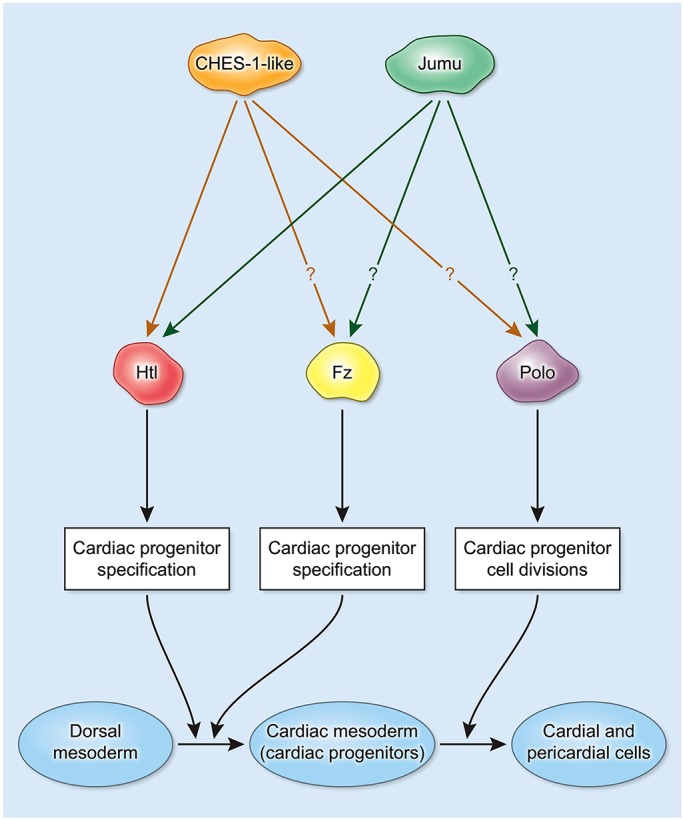
**Pleiotropic roles of *CHES-1-like* and *jumu* in cardiogenesis.** Schematic illustrating the roles of the Fkh TFs in multiple cardiogenic processes. Both CHES-1-like and Jumu transcriptionally activate expression of *htl* via its cardiac enhancer in a mutually redundant manner to achieve proper specification of cardiac progenitors from the dorsal mesoderm. Both Fkh TFs also transcriptionally activate *fz* expression to mediate CM specification, but it has yet to be determined if the activation is direct. Subsequently, both Fkh TFs activate Polo kinase to bring about proper cell divisions of these cardiac progenitors, but it is not yet known whether this activation is direct.

With the results of this study, we now show that the same Fkh genes mediate at least two distinct cardiogenic processes (Fig. 9): (1) cardiac progenitor specification by regulating the expression of the FGFR encoded by *htl* and the Wnt receptor encoded by *fz*, and (2) the subsequent cell divisions of these cardiac progenitors by regulating the activity of Polo kinase (Ahmad et al., 2012). Such pleiotropic roles for the same TFs provide an elegant illustration of how the vast array of complex processes involved in cardiogenesis can be orchestrated by a small set of regulators.

## MATERIALS AND METHODS

### *Drosophila* strains and transgenic reporter constructs

The following mutant alleles, deficiencies and transgenes were used: *Df(1)CHES-1-like1*, *Df(3R)Exel6157* and *Ubi-polo* (Ahmad et al., 2012); *twi-GAL4* (Greig and Akam, 1993); *Mef2-GAL4* (Ranganayakulu et al., 1996); *tinD-GAL4* (Yin et al., 1997); *Hand-GAL4* (Han and Olson, 2005); *UAS-2EGFP* (Halfon et al., 2002); *UAS-DN-htl* (Michelson et al., 1998b); *htl^AB42^* (Gisselbrecht et al., 1996); *stumps^YY202^* (Michelson et al., 1998a); *UAS-htl* (Michelson et al., 1998b); *fz^R52^* (Jones et al., 1996); *CHES-1-like^KK101264^* (Dietzl et al., 2007); *jumu^GL00363^* (Ni et al., 2011) and *UAS-fz1-1* (Boutros et al., 2000). Wild-type and mutated ChIPCRM2610 enhancers were synthesized by Integrated DNA Technologies, cloned into the pWattB-nlacZ vector (Busser et al., 2012) and targeted to the attP40 site (Markstein et al., 2008) by phiC31-mediated integration (Groth et al., 2004) to create the *ChIPCRM2610^WT^* and *ChIPCRM2610^Fkh^ lacZ* reporter-driving lines.

### RNA interference assays

RNA interference (RNAi) assays targeted to CM precursors were performed as previously described (Ahmad et al., 2012). UAS-RNAi constructs from the Transgenic RNAi Project (TRiP) at Harvard Medical School (Ni et al., 2011) and the Vienna *Drosophila* Resource Center (VDRC) (Dietzl et al., 2007) were expressed using both the *tinD-GAL4* and *Hand-GAL4* drivers simultaneously. The efficiency of RNAi knockdowns was enhanced both by allowing embryos to age at 29°C and by using *UAS-dcr2*.

### *In situ* hybridization, immunohistochemistry and cell counting

Embryo fixation, probe synthesis and histochemical staining were carried out as previously described (Ahmad et al., 2014; Ahmad et al., 2012; Estrada et al., 2006; Zhu et al., 2012). For all quantitative studies of gene expression, cells in >250 hemisegments were counted for each genotype examined. Because mutations in *CHES-1-like* and *jumu* cause defective cardiac progenitor cell divisions that result in certain hemisegments having atypical numbers of cells, only hemisegments with the correct number of Tin-CCs were examined and scored in *Df(1)CHES-1-like^1^* embryos, *Df(3R)Exel6157* embryos and *Df(1)CHES-1-like^1^; Df(3R)Exel6157* embryos.

### Transcriptional expression profiling

Microarray-based gene expression profiles for GFP-positive mesodermal cells isolated by fluorescence-activated sorting of single cell suspensions prepared from Stage 11-early Stage 12 *twi-GAL4 UAS-2EGFP* embryos, *Df(1)CHES-1-like^1^; twi-GAL4 UAS-2EGFP* embryos and *twi-GAL4 Df(3R)Exel6157/UAS-2EGFP Df(3R)Exel6157* embryos were obtained by Affymetrix microarray hybridization as described previously (Ahmad et al., 2012). Array data were normalized with the rma command (with default settings) in the *affy* Bioconductor package (Gautier et al., 2004) and linear models were fitted using *limma* (Smyth, 2004). The microarray data utilized in this study are available from Gene Expression Omnibus with the accession numbers GSE3854, GSE34946 and GSE65439.

### Statistical analyses

A description of the statistical analysis of genetic interaction assays, rescue assays, *cis-trans* enhancer assays and geneset enrichment is provided in the supplementary materials and methods.
